# The Role of Systemic Oxidative Status in Coronary Arterial and Peripheral Venous Blood of Patients with Unstable Angina Pectoris

**DOI:** 10.3390/life13071537

**Published:** 2023-07-11

**Authors:** Panic D. Dragan, Simic B. Ivan, Davidovic Z. Goran, Nikolic D. Maja, Lazarevic D. Nevena, Andjic M. Marijana, Vuckovic M. Jelena, Zornic J. Nenad, Zivkovic I. Vladimir, Tamara Nikolic Turnic, Jakovljevic Lj Vladimir, Iric Cupic M. Violeta

**Affiliations:** 1 Department of Cardiology, General Hospital Cuprija, Miodraga Novakovic 78, 35230 Cuprija, Serbia; 2 Department of Internal Medicine, Faculty of Medical Sciences, University of Kragujevac, Svetozara Markovica 69, 34000 Kragujevac, Serbia; 3 Department of Physiology, Faculty of Medical Sciences, University of Kragujevac, Svetozara Markovica 69, 34000 Kragujevac, Serbia; 4 Department of Pharmacy, Faculty of Medical Sciences, University of Kragujevac, Svetozara Markovica 69, 34000 Kragujevac, Serbia; 5 Department of Clinical Pharmacology, I.M. Sechenov First Moscow State Medical University (Sechenov University), 119435 Moscow, Russia; 6 Department of Cardiology, University Clinical Center Kragujevac, Zmaj Jovina 30, 34000 Kragujevac, Serbia; 7 Department of Surgery, Faculty of Medical Sciences, University of Kragujevac, Svetozara Markovica 69, 34000 Kragujevac, Serbia; 8 Center of Excellence for Redox Balance Research in Cardiovascular and Metabolic Disorders, Medical University (Sechenov University), 34000 Kragujevac, Serbia; 9 N.A. Semashko Public Health and Healthcare Department, F.F. Erismann Institute of Public Health, I.M. Sechenov First Moscow State Medical University (Sechenov University), 119991 Moscow, Russia; 10 Department of Human Pathology, University I.M. Sechenov, 1st Moscow State Medical, Trubetskaya Street 8, Str. 2, 119991 Moscow, Russia

**Keywords:** oxidative status, coronary arterial blood, peripheral venous blood, acute coronary syndrome, unstable angina pectoris

## Abstract

(1) Background: We aimed to analyze the oxidative status of patients with unstable angina pectoris (UA), as well as to determine the correlation of these parameters between coronary arterial and peripheral venous blood samples. (2) Methods: The study included 47 human subjects with UA and 45 control subjects. We performed clinical examinations, hemodynamic and coronary angiography measures. Also, in the blood samples, we measured routine laboratory markers and the concentration of pro-oxidants: index of lipid peroxidation (TBARS), superoxide anion radical (O_2_^−^), hydrogen peroxide (H_2_O_2_) and nitrites (NO_2_^−^), while antioxidant parameters were determined from red blood cells: reduced glutathione (GSH), catalase (CAT) and superoxide dismutase (SOD). All parameters were determined spectrophotometrically. (3) Results: Significantly higher values of TBARS and all measured antioxidants SOD, CAT and GSH were observed in the coronary arterial blood of the UA group relative to coronary arterial blood of the control subjects. On the other hand, in the peripheral venous blood samples, a significantly lower GSH value was found in the UA group compared to the control. (4) Conclusions: This study has shown that the majority of changes in all measured redox markers are found in coronary blood, especially related to the activity of antioxidant components. In patients with an unstable form of angina, prooxidants (superoxide anion radical and index of lipid peroxidation) and endogenous antioxidants (catalase, superoxide dismutase and reduced glutathione) are in direct correlation with the course of ischemic disease. Future studies, where participants would be randomized depending on symptom duration, are necessary to confirm these conclusions.

## 1. Introduction

During metabolic processes, potentially toxic oxygen metabolites are being formed, due to incomplete oxygen reduction, designated as reactive oxygen species (ROS). At low concentrations, ROS plays an important role in physiological systems. From the aspect of atherogenesis, ROS are modulated by several vascular cell functions, including endothelial and smooth muscle cell growth, proliferation and migration, angiogenesis, apoptosis, vascular tone, immune capacity and genomic stability [1,2]. The normal functioning of the cell requires a balance between ROS and neutralizing antioxidants. When ROS production exceeds antioxidant defense capacity, a condition labeled as oxidative stress with consequent cell damage occurs. The degree of damage is determined by the target structure as well as by the ROS concentration [3]. Oxidative stress could be one of the key factors in the occurrence of endothelial dysfunction and a common denominator of many proatherogenic stimuli such as dyslipidemia, hypertension, diabetes, smoking, hyperhomocysteinemia, infection and inflammation [4].

Over the last few decades, research has increasingly suggested the universality of existing oxidative stress in various pathological conditions [4]. The pathogenic basis of unstable angina pectoris (UA), as a form of acute coronary syndrome, is usually the destabilization of the previously stable atherosclerotic plaque. The process of atherogenesis leading to unstable atherosclerotic plaque has not been fully elucidated, but there is increasing evidence that oxidative stress and inflammation are associated with atherosclerotic plaque instability and the occurrence of acute coronary syndrome (ACS) [1,4]. 

Increased ROS production has a strong influence on the basic stages of atherogenesis such as LDL oxidation, endothelial and smooth muscle cell dysfunction, as well as on monocyte growth and migration. Oxidative stress modulates gene expression in endothelial and smooth muscle cells through enhanced production of oxidized LDL particles [5]. Additionally, oxidative stress increases the activity of various members of the family of mitogen-activating protein kinases (MAP kinases), tyrosine kinases and protein kinases isoforms. These enzymes are involved in the activation of various transcription factors that directly affect the activity of genes in the target cell, such as genes for adhesive molecules (ICAM-1, VCAM-1, E selectin and P selectin), as well as a gene for the monocyte attracting protein, MCP-1 (monocyte chemotactic protein-1) [5,6,7].

Taking into account that the link between oxidative stress and the pathogenic basis of processes involved in UA truly exists, attention has been on the rise for the identification of oxidative markers that can serve as predictors of plaque destabilization and the occurrence of unwanted coronary events. However, data from experimental and clinical studies are still poor and inconsistent followed by many limitations and biases [8,9,10]. Therefore, until now there is no concrete success in this direction. Oxidative stress plays a key role in ischemia-reperfusion injury [8,9,10,11] and subsequent cardiac repair. Interruption of blood flow in the coronary arteries causes ischemia of adjacent tissues, leading to cell injury, which may result in cell necrosis and apoptosis. The duration of ischemia determines the extent of damage to the myocardium and related tissues [12]. During ischemia, cellular defenses against oxidative injury are impaired, with lower activities of antioxidants such as superoxide dismutase and glutathione peroxidase. Furthermore, greater amounts of ROS are produced, for example, by xanthine dehydrogenase, which is converted to xanthine oxidase, a potent generator of O_2_^−^ and hydrogen peroxide [13,14,15,16,17]. Concerning the detection of specific redox markers in patients with UA seems to be difficult from this point [18,19], as we seek to find a correlation between oxidative stress parameters in peripheral blood and the same in the coronary blood of these patients. Our hypothesis was that there is a mutual relationship that could help in risk stratification and treatment of UA.

Therefore, the present investigation aimed to analyze parameters of oxidative stress and antioxidative defense in patients with unstable angina pectoris (UA), in blood samples collected from the coronary artery and blood samples collected from peripheral venous blood, as well as to determine the correlation of these parameters between arterial and venous blood. Specifically, this study aimed to find valuable predictors of unstable angina from the range of biomarkers of oxidative stress.

## 2. Materials and Methods

### 2.1. Ethic Statement

This research was conducted in accordance with the principles outlined in the Declaration of Helsinki and the principles of Good Clinical Practice (GCP). The study protocol was approved by the Ethics Committee of the Clinical Center Kragujevac before the onset of the study (date of approval 21 January 2017). Before all study procedures, written informed consent was provided by all the participants of the study.

### 2.2. Patients

The study involved patients hospitalized at the Clinic for Cardiology, Clinical Center Kragujevac, Serbia. A total of 92 subjects who met the inclusion criteria were included in the study and divided into experimental and control groups. Forty-seven patients with unstable angina belong to the experimental UA group, while the control group included 45 patients of similar sex and age distributions who were found to have angiographically completely pure coronary blood vessels after selected coronary angiography. The patients who were shown to have stenosis diameter >50% after angiography were included in the experimental group at the Emergency Department of Clinical Center Kragujevac. The main criteria for inclusion in the study were diagnosis of unstable angina pectoris and normal troponin I value at admission. Other criteria were signed informed written consent for participating in the study. The inclusion criteria for the control group subjects were a negative finding on the cardiology examination, absence of any symptoms and previous cardiovascular disorders.

The exclusion criteria were: patients with troponin I enzyme above referent values, patients under 18 and older than 80 years, pregnancy, impaired cognitive state, patients with postinfarction angina pectoris (first two weeks after infarction), surgically revascularized patients, patients treated with nebivolol and nitrate preparations, patients on hemodialysis and patients with impaired thyroid function (Figure S1). 

### 2.3. Blood Sampling and Laboratory Test in Study Groups

After clinical processing of patients (anamnestic data with identification of risk factors, laboratory analysis and echocardiographic examination), all patients underwent coronary angiography. Laboratory measurement consists of a list of routine laboratory markers. On the first day of hospital admission, samples of peripheral venous blood were drawn from the antecubital vein after patient overnight fasting, and processed for a complete series of routine laboratory assays. The laboratory variables explored were hematocrit, white blood cell (WBC) count, neutrophil-to-lymphocyte (N/L) ratio, platelet count, fasting glucose, serum creatinine, total cholesterol, high-density lipoprotein cholesterol (HDLc), triglycerides and C-reactive protein. Low-density lipoprotein cholesterol (LDLc) concentration was calculated using the Friedewald equation. The glomerular filtration rate (eGFR) was estimated according to the Cockcroft–Gault formula.

In subjects with both angiographically pure blood vessels and with unstable atherosclerotic plaque, coronary arterial blood was taken from the right coronary artery by using a coronary catheter. Immediately after the sample of coronary arterial blood was taken, a sample of peripheral venous blood was also taken from the cubital non-dominant hand vein for the planned laboratory analysis. Blood was collected in vacutainer tubes containing 0.129 M sodium citrate (BD Vacutainer Blood Collection System) using a 21-gauge polyethylene catheter for taking blood samples (BD Vacutainer needles). Blood was centrifuged to separate plasma and red blood cells (RBCs) and stored at −20 °C until oxidative stress analyses [20].

### 2.4. Oxidative Stress Evaluating Using Spectrophotometric Method 

All the substances used for the analyses of oxidative stress parameters were purchased from Sigma-Aldrich, Germany.

The oxidative stress parameters were measured spectrophotometrically (apparatus UV-1800, Shimadzu, Japan). We measured the concentration of the following pro-oxidative markers in plasma samples: hydrogen peroxide (H_2_O_2_), superoxide anion radical (O_2_^−^), nitrites (NO_2_^−^) and index of lipid peroxidation measured as TBARS (thiobarbituric acid reactive substances). The concentration of reduced glutathione (GSH) and the activity of the enzymatic defense system (CAT—catalase, SOD—superoxide-dismutase) were determined from RBC.

### 2.5. Determination of Plasma Prooxidant Parameters (TBARS, NO_2_^−^, O_2_^−^, H_2_O_2_)

The degree of lipid peroxidation in plasma samples was estimated by measuring thiobarbituric acid reactive substances (TBARS) according to Ohkawa H, et al. [21]. We used 0.4 mL 1% thiobarbituric acid (TBA) in 0.05 NaOH mixed with 0.8 mL of plasma, incubated at 100 °C for 15 min and measured at 530 nm. Distilled water was used as a blank probe. TBA extract was obtained by combining 0.8 mL plasma and 0.4 mL TCA (trichloroacetic acid). Afterward, samples were put on ice for 10 min, and centrifuged for 15 min at 6000 rpm [21].

Nitric oxide (NO) decomposes rapidly to form stable metabolite nitrite/nitrate products. The method for detection of the plasma nitrite levels is based on the Griess reaction. Nitrites (NO_2_^−^) were determined as an index of NO production with Griess reagent (forms purple diazocomplex). An amount of 0.1 mL 3 N PCA (perchloric acid), 0.4 mL 20 mM EDTA (ethylenediaminetetraacetic acid) and 0.2 mL plasma were put on ice for 15 min, then centrifuged for 15 min at 6000 rpm. After pouring off the supernatant, 220 μL K_2_CO_3_ was added. Nitrites were measured at 550 nm. Distilled water was used as a blank probe [22].

The level of superoxide anion radical (O_2_^−^) was measured using Nitro Blue Tetrazolium (NBT) reaction in TRIS buffer with plasma and read at 550 nm. Distilled water was used as a blank probe [23].

Determination of hydrogen peroxide (H_2_O_2_) concentration is based on the oxidation of phenol red using hydrogen peroxide, in a reaction catalyzed by enzyme peroxidase from horse radish (POD). A 200 μL sample with 800 μL PRS (phenol red solution) and 10 μL POD were combined (1:20) and measured at 610 nm [24].

### 2.6. Determination of Hemolysate Antioxidant Parameters (CAT, SOD, GSH)

Isolated RBCs were washed three times with 3 volumes of ice-cold 0.9 mmol/L NaCl and hemolysates containing about 50 g Hb/L, prepared according to McCord and Fridovich [25], were used for the determination of catalase (CAT) activity. Determination of CAT activity was determined according to Beutler [26]. Lysates were diluted with distilled water (1:7 *v*/*v*) and treated with chloroform–ethanol (0.6:1 *v*/*v*) to remove hemoglobin. Then, 50 μL CAT buffer, 100 μL sample and 1 mL 10 mM H_2_O_2_ were added to the samples. Detection was performed at 360 nm. Distilled water was used as a blank probe. 

Determination of superoxide dismutase (SOD) activity is based on the epinephrine method of Misra and Fridovich [27]. A 100 μL lysate and 1 mL carbonate buffer were mixed, and then epinephrine in a volume of 100 μL was added. Detection was performed at 470 nm. This method belongs to the ’negative’ type group of methods, since it monitors the decrease in autoxidation speed in an alkaline medium, which is dependent on O_2_^−^. 

The level of reduced glutathione (GSH) concentration was determined based on GSH oxidation with 5.5-dithiobis-6.2-nitrobenzoic acid, using the Beutler method [27]. Measurement of the absorbance is carried out at a wavelength of maximum absorption of 420 nm.

### 2.7. Statistical Analysis

All data are presented as mean ± standard deviations or as a frequency in percent (%). Statistical significance was estimated using Student’s *t*-test with statistical significance at the level *p* < 0.05 between healthy subjects and unstable angina patients. Linear regression model was used to test the significant predictor in a negative outcome. All analysis was done in the licensed statistical program SPSS version 26 (IBM SPSS, New York, NY, USA for Macintosh). 

## 3. Results

### 3.1. Basic Characteristics of Study Population

This study included 92 subjects with an age of 63.95 ± 8.34 years. In the UA group, the age of the subjects was 64.79 ± 7.99 years, while the age of the subjects in the control group was 63.07 ± 8.69 years. All the baseline demographic and socio-epidemiological data of the study population are presented in the form of Table 1. 

### 3.2. Clinical Parameters of Participants in Study Groups 

In both groups, we measured systolic and diastolic blood pressure (BP) at admission, as well as ejection fraction (EF). There were no statistically significant differences between these parameters in CTRL and UA groups (Table 2).

### 3.3. Laboratory Parameters of Healthy and Participants with UA

In the form of Table 3, we presented the levels of routine laboratory markers in healthy and participants measured at admission. We observed only statistical significance in the term of triglycerides, where the patients with UA had higher levels in comparison with healthy participants.

### 3.4. Concentrations of Plasma Pro-Oxidative Markers in Study Population

**Figure 1:**
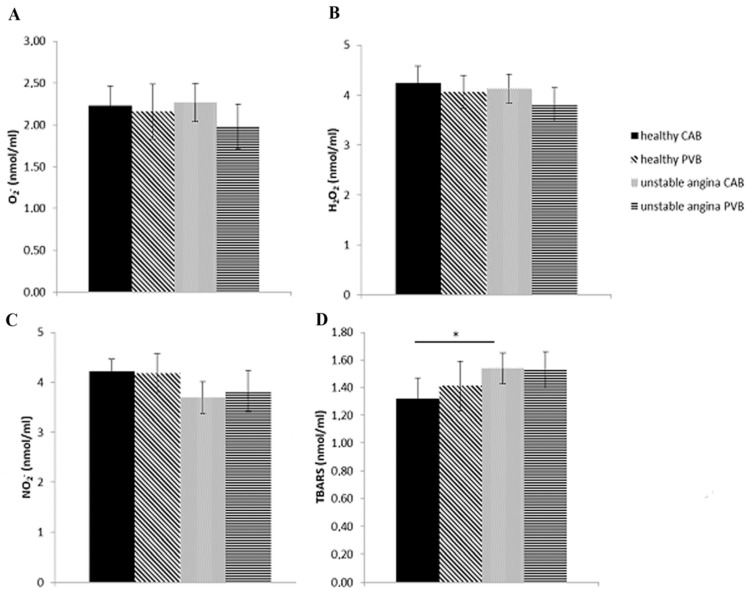
The values of pro-oxidant parameters: (**A**) superoxide anion radical (O_2_^−^); (**B**) hydrogen peroxide (H_2_O_2_); (**C**) nitrites (NO_2_^−^); (**D**) index of lipid peroxidation in the form of TBARS (thiobarbituric acid reactive substances) in the plasma of healthy and unstable angina patients. * statistical significance at the level *p* < 0.05 between healthy subjects and unstable angina patients. CAB: coronary arterial blood; PVB: peripheral venous blood. Data are presented as means ± SD.

There were no significant differences in the values of pro-oxidant parameters, between blood samples taken from the coronary artery and peripheral vein, and neither in the control group nor in the experimental group of unstable angina patients. Additionally, no significant differences in the values of O_2_^−^, H_2_O_2_ and NO_2_^−^ were noticed between the groups, when comparing healthy and unstable angina patients both in coronary and venous blood samples. On the other hand, the index of lipid peroxidation measured as TBARS was found to be significantly increased (*p* = 0.035) in the coronary arterial blood samples in patients with unstable angina relative to healthy controls (Figure 1). 

### 3.5. Antioxidant Enzyme Activity in Study Population

**Figure 2:**
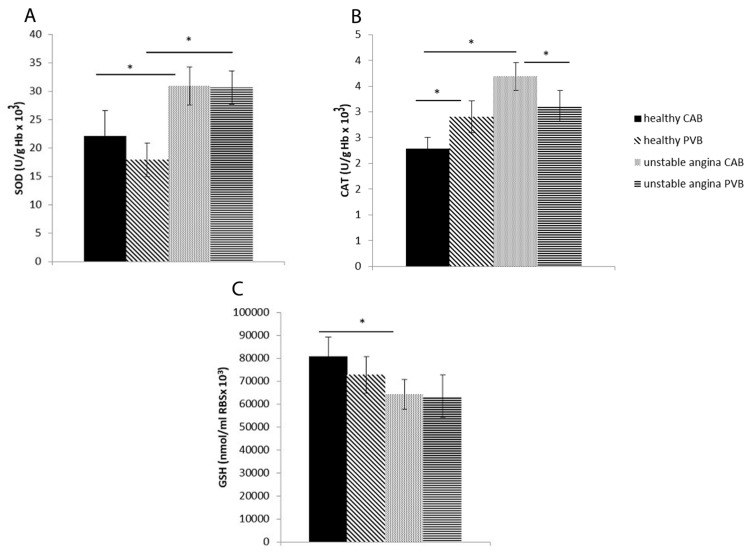
The values of antioxidant parameters. The activity of (**A**) superoxide dismutase (SOD); (**B**) catalase (CAT); (**C**) the values of reduced glutathione (GSH) in the RBCs of healthy and unstable angina patients. * statistical significance at the level *p* < 0.05 between healthy subjects and unstable angina patients. CAB: coronary arterial blood; PVB: peripheral venous blood. Data are presented as means ± SD.

Antioxidant enzymes SOD and CAT were significantly increased in patients with unstable angina pectoris compared to their healthy controls in coronary arterial blood samples, while the significant elevation of SOD in the experimental group was also noticed in peripheral venous blood samples. The level of GSH was significantly lowered (*p* = 0.016) in the coronary arterial blood samples of patients with UA relative to CTRL. No significant differences within the groups (between arterial and venous blood) were observed in antioxidant parameters, except for SOD (Figure 2).

### 3.6. Regression Analysis of Data 

We used correlation analysis to check the potential association of all variables. Based on that correlation data, we used the univariate regression model to calculate the predictors of unstable angina in patients using the significantly correlated variables (concentrations of all biomarkers of oxidative stress). As shown in the form of Table 1, the significant predictors are superoxide anion radical, nitric oxide and index of lipid peroxidation. Also, lower levels of superoxide dismutase, catalase and reduced glutathione were observed as negative predictors of unstable angina (Table 4).

## 4. Discussion

To the best of our knowledge, this is the first study comparing oxidative stress between a coronary artery and peripheral venous blood samples, although there have been other investigations comparing basal ROS production in arteries and veins with contradictory conclusions [18]. Shi et al. showed a higher production of superoxide anion radical in venous compared to arterial grafts in pig models, as well as a lower level of SOD in venous compared to arterial grafts [22]. However, Guzik et al. did not find a significant difference in the production of superoxide anion radical between the arteria mammaria interna and the *vein saphena* in man, which is in line with our findings regarding O_2_^−^ [23]. Others claimed the opposite, they found that the superoxide anion radical was increased in the inferior cavity vein relative to the aorta and also was higher in the mesenteric vein compared to the artery of the same name [24].

Several studies have compared oxidative status in the venous coronary sinus and the peripheral vein. In their study, Lantos showed that in early blood samples, first 60 min of reperfusion, taken from the coronary sinus, after 60 min ischemia, there was a significantly higher concentration of MDA and SOD and lower GSH compared to the values of these parameters obtained from the peripheral venous blood of dogs. However, the authors of the study found that values of these parameters obtained from a peripheral vein sample and after 60 min of reperfusion, are also informative and can be taken to evaluate the severity of oxidative stress [25]. In assessing myocardial metabolic activity, blood samples obtained from the coronary sinus take precedence over peripheral venous blood samples, although the same authors state that the method is limited by the complexity and invasiveness of the procedure, the use of contrast agents, the appearance of supraventricular arrhythmias and difficulty in maintaining a stable position and also catheters and serial measurements over time are impractical [26].

According to our results, generally, there was no difference noticed neither in the release of oxidative stress parameters between coronary blood samples and peripheral venous blood samples within the UA group nor in the control group. More specifically, except for TBARS, pro-oxidants did not show changes between arterial and vein blood or UA and healthy patients. Unlike this, antioxidant enzymes showed interesting dynamics in coronary blood when comparing UA and the control group.

In the present research, analysis of blood samples collected from the coronary artery revealed a difference in TBARS values between the UA group and the CTRL group. This is in line with previous findings that oxidative stress and lipid peroxidation are fundamental to the process of atherogenesis. The TBARS method is a sensitive method for the determination of lipid peroxidation, which is based on the reaction of malonylaldehyde (MDA) with two thiobarbituric acid molecules that produce a complex that can be measured spectrophotometrically [5]. MDA is a terminal product of oxidative damage to polyunsaturated fatty acids and is commonly used as a marker of lipid peroxidation in analytical methods [27,28]. The link between oxidative stress expressed through MDA and acute coronary syndrome has been the subject of numerous studies. The previous study indicates that in patients with coronary artery disease, MDA was significantly elevated and antioxidant status was significantly reduced compared to the control group of healthy subjects [29].

The role of lipid peroxidation and MDA in coronary disease and the ability to differentiate unstable from a stable form of disease have been the subject of numerous clinical studies. The study by McMurray et al. showed that the mean level of TBARS was significantly elevated in the patient population with UA compared to the CTRL group of healthy subjects and it was possible to differentiate unstable angina from stable angina based on plasma thiol values [30]. Also, plasma MDA levels in patients with unstable angina and acute myocardial infarction (MI) were higher than those in subjects with stable angina pectoris as in healthy volunteers. This study did not show differences in MDA and GSH values between subjects with UA and MI [26]. A rise in the level of total MDA was observed in the unstable angina group and stable angina group compared to the control group, with higher levels of free MDA differentiating the UA from stable angina [31]. This finding is similar to the results of other research which showed that the level of MDA was significantly higher in subjects with UA relative to stable angina and the level of reduced glutathione was significantly lower in subjects with unstable angina than in SA (*p* < 0.001). In the same study, no differences were observed between UA and MI subjects [32].

Data in the literature indicate that attempts to define the complexity of lesions via the MDA value. Yilmaz et al. demonstrated that MDA in stable coronary artery disease may differentiate coronary artery disease but not the extent and complexity expressed by the SYNTAX score [33]. This finding is consistent with the results of a study that also indicated that oxidative stress may reflect the intensity but not the complexity of coronary artery disease [34].

The increased degree of lipid peroxidation and the decreased level of reduced glutathione detected in our study are in correlation with the data in the literature. In the peripheral venous blood samples, there was a statistically significant increase in GSH in the control group compared to the unstable angina group. Glutathione status is important information on oxidative events in the cell. Reduced glutathione in reaction with peroxides transforms into the oxidized form of glutathione (GSSG), which prevents oxidation of cell membrane lipids. In UA in the zone of unstable atherosclerotic plaque, increased oxidative stress also causes increased consumption of reduced glutathione, which is reflected in its reduced values in the peripheral blood.

The level of GSH was significantly decreased in patients with AMI, while MDA levels were elevated in patients with AMI compared with those in the control group [35]. The study on 80 subjects showed no difference in the reduced glutathione values between the subjects with stable angina, unstable angina or MI, as well as the healthy controls. Only glutathione reductase activity was significantly higher in IM subjects compared to SA [36]. Also, the levels of glutathione reductase in the platelets of unstable angina patients are reduced compared to healthy subjects, which would indicate a reduced antioxidant capacity of reduced glutathione and a reduced ability to remove hydrogen peroxide in a patient with unstable angina [37]. Some results showed significantly higher glutathione reductase levels in UA patients, which gradually decreased with disease stabilization. During the six-month follow-up, the subjects maintained low levels of glutathione reductase without new cardiovascular events. Thus, the authors conclude that the activity of this enzyme was an independent predictor of cardiovascular events during the follow-up period [38].

In the present research, a difference in CAT and SOD activity between the UA and the control group was obtained from the coronary arteries. Higher activities of both enzymes in coronary blood samples of patients with unstable angina can be explained by a compensatory mechanism that in response to oxidative stress in the early stages corresponds to the increased production of this enzyme. However, in the later stages when free radicals reach a chronically elevated level, this mechanism becomes insufficient and shows a decrease in the activity of antioxidant enzymes [35]. Based on these results, it can be hypothesized that increased activation of SOD and CAT in coronary arteries of UA patients can be responsible for the absence of difference between the release of O_2_^−^ and H_2_O_2_ compared to the control.

Our finding is consistent with the study which showed that in the early stages of coronary atherosclerosis, SOD and CAT levels were increased and significantly were worsening with disease progression [39]. Analyzing SOD values, our study did not show a difference within the groups between the arterial and peripheral venous blood samples. Similar results were found in a previous investigation that did not register a statistically significant difference in extracellular SOD values between control subjects and coronary patients [40]. However, decreased SOD activity was also found in patients with coronary artery disease, with a significant difference between stenosis greater than 50% compared to stenosis less than 50%. In the same study, there was no significant difference in SOD values between subjects with stenosis less than 50% and the control group [38].

Definitely, in clinical practice, there are still traditional predictors of UA which are followed by clinicians. Measurement of a biomarker of necrosis is recommended for all patients with suspected ACS. On the basis of tissue specificity and sensitivity, cardiac troponin (cTn) is the preferred biomarker of necrosis for diagnosis and risk stratification [41]. The detection of cTn in the peripheral circulation is indicative of myocardial injury and in patients with a clinical syndrome consistent with ACS is associated with greater coronary lesion complexity, more frequent visible thrombus, more severely impaired flow in the culprit artery and diminished microvascular perfusion. An elevated cTn is associated with a ≈ 4-fold higher risk of death or MI among patients with ACS. Improvements in the analytic performance of available assays, in conjunction with evidence confirming the clinical relevance of low concentrations of cTn, have supported professional recommendations to use a single cut point at the 99th percentile of a reference population as the decision limit for risk stratification. In patients with stable CHD, biochemical evaluation of total cholesterol, low-density lipoprotein (LDL) cholesterol, high-density lipoprotein cholesterol, triglycerides, serum creatinine (estimated glomerular filtration) and fasting blood glucose is important to guide preventive interventions recommended by current professional guidelines [42,43]. 

On the contrary, more specific markers in the prediction of diseases mediated by oxidative stress are systemic levels of endogenous pro-oxidants and antioxidants. Our study confirmed the significant role of redox status biomarkers in the prediction of ischemic attacks. There are some advantages of these markers in prediction: for example, the natriuretic peptide could not be a marker of unstable angina; cardiac troponins are not still the mainstream in clinical practice because of the low potential to predict prognosis and mortality; cholesterol and triglyceride levels are the markers of atherosclerosis but not specific for coronary disease; and lipoprotein has a low predictive value for heart failure in general. All these defects of the potential of conventional markers need to find more specific markers in predicting ischemic disease. Various studies conducted in the last few decades, which have focused on markers of oxidative stress in plasma and tissues of patients with atherothrombosis, have often had ambiguous results [6,44]. The results of our study are in agreement with other research, but, on the other hand, there were also studies with results more or less different from ours. Although some indicators of oxidative stress predict increased cardiovascular risk, for the time being, there is no clinically valid, specific biomarker for patients with coronary disease, as biomarkers studied so far are not closely disease-specific but reflect biological activity associated with pathology [6,45,46,47,48,49]. Although there have been promising results in recent years, methodologically appropriate, prospective research is needed to clarify this problem in the future.

The limitation of this study lies in the short period of following the patients. Based on the inclusion criteria, we used a small homogenous group of patients and matched controls but in the short-term following. That was the unique way to be sure and exclude potential bias in every obtained parameter and patient data during the study. In the future, studies need to take longer periods for estimating the dynamic of oxidative stress. Also, it is recommended that further studies include antioxidant therapy as an adjuvant in order to find potential preventive strategies in UA patients. 

## 5. Conclusions

This study has shown that the majority of changes in all measured redox markers are found in coronary blood, especially related to the activity of antioxidant components. In patients with an unstable form of angina, prooxidants (superoxide anion radical and index of lipid peroxidation) and endogenous antioxidants (catalase, superoxide dismutase and reduced glutathione) are in direct correlation with the course of ischemic disease. Future studies, where participants would be randomized depending on symptom duration are necessary to confirm these conclusions.

## Figures and Tables

**Table 1:** Basic demographic and socio-epidemiological data of study population. Statistical significance was estimated using Student’s *t*-test with statistical significance at the level *p* < 0.05 between healthy subjects (CTRL) and unstable angina (UA) patients.

Variable	CTRL Group [n = 45]	UA Group [n = 47]	*p* Value
Gender (F/M) per group [%] Gender per study population [%]	F 20 (44.4) M 25 (55.6)	F 18 (38.3) M 29 (61.7)	*p* < 0.05
	F 38 (35.9)	M 54 (64.1)	*p* < 0.05
Age (years)	63.07 ± 8.69	64.79 ± 7.99	*p* > 0.05
Hypertension [%]	24.2	22.5	*p* > 0.05
Dyslipidemia [%]	4.45	6.23	
Smoking [%]	10.2	9.4	
Diabetes Mellitus [%]	3.7	23.4	*p* < 0.05
Previous vascular events [%]	4.4	42.6	
Previous percutaneous coronary intervention [%]	8.9	12.8	

**Table 2:** Systolic, diastolic blood pressure (BP) and ejection fraction (EF) in CTRL and UA groups at admission. Statistical significance was estimated using Student’s *t*-test with statistical significance at the level *p* < 0.05 between healthy subjects and unstable angina patients.

Variable	CTRL Group [n = 45]	UA Group [n = 47]	*p* Value
Systolic BP [mmHg]	127.88 ± 20.33	132.12 ± 15.77	*p* > 0.05
Diastolic BP [mmHg]	74.72 ± 12.06	78.63 ± 9.27	
EF [%]	53.55 ± 11.11	52.21 ± 9.21	

**Table 3:** Routine laboratory markers in healthy and participants with UA. Statistical significance was estimated using Student’s *t*-test with statistical significance at the level *p* < 0.05 between healthy subjects (CTRL) and unstable angina (UA) patients.

Variable	CTRL Group [n = 45]	UA Group [n = 47]	*p* Value
Hematocrit, mean (SD), (%)	40.31 ± 5.21	40.12 ± 6.22	*p* > 0.05
White blood cells/mm^3^, mean (SD)	7.55 ± 2.25	7.42 ± 2.22	
Neutrophil/lymphocyte ratio, mean (SD)	2.67 ± 1.10	7.73 ± 1.13	
Platelets/mm^3^, mean (SD)	223.34 ± 64.23	226.87 ± 67.09	
Glucose, mean (SD), (mg/dL)	111.56 ± 37.21	107.23 ± 36.41	
Creatinine, mean (SD), (mg/dL)	1.01 ± 0.33	1.11 ± 0.21	
Total cholesterol, mean (SD), (mg/dL)	180.23 ± 43.21	185. 44 ± 32.12	
High-density cholesterol, mean (SD), (mg/dL)	39.87 ± 12.87	40.45 ± 10.09	
Low-density cholesterol, mean (SD), (mg/dL)	116.77 ± 13.78	119.22 ± 32.12	
Triglycerides, mean (SD), (mg/dL)	124.66 ± 58.21	137.34 ± 67.12	*p* < 0.05 *
C-reactive protein, mean (SD), (mg/dL)	1.11 ± 2.83	0.99 ± 2.11	*p* > 0.05

* represent the statistical significance below 0.05.

**Table 4:** Regression analysis on the predictor of unstable angina.

Variable	B	Std. Error	Beta	*t* Value	*p* Value
Superoxide anion radical [nmol/L]	18.812	3.637	0.355	5.173	0.034
Hydrogen peroxide [nmol/L]	0.263	0.042	0.211	6.318	0.051
Nitric oxide [nmol/L]	0.178	0.001	0.231	4.321	0.011
Index of lipid peroxidation [micromol/L]	17.12	2.043	0.111	2.341	0.023
Superoxide dismutase [U/Hg × 10^9^]	1.789	0.143	0.546	−6.440	0.001
Catalase [U/Hg × 10^9^]	3.789	0.301	0.234	−5.431	0.001
Reduced glutathione [U/Hg × 10^9^]	4.121	1.212	0.398	−2.341	0.111

Note: R = 0.59, R2 = 0.36, Adj.R2 = 0.35.

## Data Availability

All data are available on request from the corresponding author.

## Supplementary Materials

The following supporting information can be downloaded at: https://www.mdpi.com/article/10.3390/life13071537/s1, Figure S1: CONSORT flow diagram. CONSORT, Consolidated Standards of Reporting Trials.

## References

[1] Vichova T., Motovska Z. (2013). Oxidative stress: Predictive marker for coronary artery disease. Exp. Clin. Cardiol..

[2] Münzel T., Camici G.G., Maack C., Bonetti N.R., Fuster V., Kovacic J.C. (2017). Impact of Oxidative Stress on the Heart and Vasculature: Part 2 of a 3-Part Series. J. Am. Coll. Cardiol..

[3] Gracia K.S., Llanas-Cornejo D., Husi H. (2017). CVD and Oxidative Stress. J. Clin. Med..

[4] Senoner T., Dichtl W. (2019). Review Oxidative Stress in Cardiovascular Diseases: Still a Therapeutic Target?. Nutrients.

[5] Đukić M. (2008). Lipidna peroksidacija indukovana slobodnim radikalima. Oksidativni Stres: Kliničko-Dijagnostički Značaj.

[6] Martin-Ventura J.L., Rodrigues-Diez R., Martinez-Lopez D., Salaices M., Blanco-Colio L.M., Briones A.M. (2017). Oxidative Stress in Human Atherothrombosis: Sources, Markers and Therapeutic Targets. Int. J. Mol. Sci..

[7] Marchio P., Guerra-Ojeda S., Vila J.M., Aldasoro M., Victor V.M., Mauricio M.D. (2019). Targeting Early Atherosclerosis: A Focus on Oxidative Stress and Inflammation. Oxid. Med. Cell. Longev..

[8] Ehara S., Ueda M., Naruko T., Haze K., Itoh A., Otsuka M., Komatsu R., Matsuo T., Itabe H., Takano T. (2001). Elevated levels of oxidized low density lipoprotein show a positive relationship with the severity of acute coronary syndromes. Circulation.

[9] Ferrari R., Guardigli G., Mele D., Percoco G.F., Ceconi C., Curello S. (2004). Oxidative stress during myocardial ischaemia and heart failure. Curr. Pharm. Des..

[10] Zhao Z.Q., Vinten-Johansen J. (2002). Myocardial apoptosis and ischemic preconditioning. Cardiovasc. Res..

[11] Naruko T., Ueda M., Ehara S., Itoh A., Haze K., Shirai N., Ikura Y., Ohsawa M., Itabe H., Kobayashi Y. (2006). Persistent high levels of plasma oxidized low-density lipoprotein after acute myocardial infarction predict stent restenosis. Arterioscler. Thromb. Vasc. Biol..

[12] Nagayoshi Y., Kawano H., Hokamaki J., Miyamoto S., Kojima S., Shimomura H., Tsujita K., Sakamoto T., Yoshimura M., Ogawa H. (2005). Urinary 8-hydroxy-2′-deoxyguanosine levels increase after reperfusion in acute myocardial infarction and may predict subsequent cardiac events. Am. J. Cardiol..

[13] Siwik D.A., Colucci W.S. (2004). Regulation of matrix metalloproteinases by cytokines and reactive oxygen/nitrogen species in the myocardium. Heart Fail. Rev..

[14] Keith M., Geranmayegan A., Sole M.J., Kurian R., Robinson A., Omran A.S., Jeejeebhoy K.N. (1998). Increased oxidative stress in patients with congestive heart failure. J. Am. Coll. Cardiol..

[15] Kaneda H., Taguchi J., Ogasawara K., Aizawa T., Ohno M. (2002). Increased level of advanced oxidation protein products in patients with coronary artery disease. Atherosclerosis.

[16] Azumi H., Inoue N., Ohashi Y., Terashima M., Mori T., Fujita H., Awano K., Kobayashi K., Maeda K., Hata K. (2002). Superoxide generation in directional coronary atherectomy specimens of patients with angina pectoris: Important role of NAD(P)H oxidase. Arterioscler. Thromb. Vasc. Biol..

[17] Ide T., Tsutsui H., Hayashidani S., Kang D., Suematsu N., Nakamura K., Utsumi H., Hamasaki N., Takeshita A. (2001). Mitochondrial DNA damage and dysfunction associated with oxidative stress in failing hearts after myocardial infarction. Circ. Res..

[18] Arroyo C.M., Kramer J.H., Dickens B.F., Weglicki W.B. (1987). Identification of free radicals in myocardial ischemia/reperfusion by spin trapping with nitrone DMPO. FEBS Lett..

[19] Kurian G.A., Rajagopal R., Vedantham S., Rajesh M. (2016). The Role of Oxidative Stress in Myocardial Ischemia and Reperfusion Injury and Remodeling: Revisited. Oxid Med Cell Longev..

[20] https://www.thermofisher.com/rs/en/home/references/protocols/cell-and-tissue-analysis/elisa-protocol/elisa-sample-preparation-protocols/plasma-and-serum-preparation.html.

[21] Ohkawa H., Ohishi N., Yagi K. (1979). Assay for lipid peroxides in animal tissues by thiobarbituric acid reaction. Anal. Biochem..

[22] Green L.C., Wagner D.A., Glogowski J., Skipper P.L., Wishnok J.S., Tannenbaum S.R. (1982). Analysis of nitrate, nitrite, and [15N]nitrate in biological fluids. Anal. Biochem..

[23] Auclair C., Voisin E., Greenwald R.A. (1985). Nitroblue tetrazolium reduction. Handbook of Methods for Oxygen Radical Research.

[24] Pick E., Keisari Y. (1980). A simple colorimetric method for the measurement of hydrogen peroxide produced by cells in culture. J. Immunol. Meth..

[25] Beutler E. (1982). Catalase. Red Cell Metabolism, a Manual of Biochemical Methods.

[26] Misra H.P., Fridovich I. (1972). The role of superoxide-anion in the autoxidation of epinephrine and a simple assay for superoxide dismutase. J. Biol. Chem..

[27] Beutler E. (1975). Reduced glutathione (GSH). Red Cell Metabolism, a Manual of Biochemical Methods.

[28] Szasz T., Thakali K., Fink G.D., Watts S.W. (2007). A comparison of arteries and veins in oxidative stress: Producers, destroyers, function, and disease. Exp. Biol. Med..

[29] Shi Y., Patel S., Davenpeck K.L., Niculescu R., Rodriguez E., Magno M.G., Ormont M.L., Mannion J.D., Zalewski A. (2001). Oxidative stress and lipid retention in vascular grafts: Comparison between venous and arterial conduits. Circulation.

[30] Guzik T.J., Sadowski J., Kapelak B., Jopek A., Rudzinski P., Pillai R., Korbut R., Chanon K.M. (2004). Systemic regulation of vascular NAD(P)H oxidase activity and nox isoform expression in human arteries and veins. Arterioscler. Thromb. Vasc. Biol..

[31] Lantos J., Temes G., Göbölös L., Jaberansari M.T., Roth E. (2001). Is peripheral blood a reliable indicator of acute oxidative stress following heart ischemia and reperfusion?. Med. Sci. Monit..

[32] Jaumdally R., Varma C., Macfadyen R.J., Lip G.Y.H. (2007). Coronary sinus blood sampling: An insight into local cardiac pathophysiology and treatment?. Eur. Heart J..

[33] Bhat M.A., Mahajan M., Gandhi G. (2012). Oxidative stress status in coronary Artery disease patients. Int. J. Life Sci. Biotechnol. Pharma Res..

[34] Thakur K.S., Jaggi K., Rathore B., Chander R., Mahdi F., Mathur A. (2014). Assessment of oxidative stress, antioxidant enzymes and lipid profile in the subjects of coronary artery disease (CAD). Int. J. Pharm. Sci..

[35] McMurray J., Chopra M., Abdullah I., Smith W.E., Dargie H.J. (1992). Evidence for oxidative stress in unstable angina. Br. Heart J..

[36] Dubois-Randé J.L., Artigou J.Y., Darmon J.Y., Habbal R., Manuel C., Tayarani I., Castaigne A., Grosgogeat Y. (1994). Oxidative stress in patients with unstable angina. Eur. Heart J..

[37] Cavalca V., Cighetti G., Bamonti F., Loaldi A., Bortone L., Novembrino C., De Franceschi M., Belardinelli R., Guazzi M.D. (2001). Oxidative stress and homocysteine in coronary artery disease. Clin. Chem..

[38] Uppal N., Uppal V., Uppal P. (2014). Progression of Coronary Artery Disease (CAD) from Stable Angina (SA) towards Myocardial Infarction (MI): Role of Oxidative Stress. J. Clin. Diagn. Res..

[39] Yılmaz M., Altın C., Özyıldız A., Müderrisoğlu H. (2017). Are oxidative stress markers helpful for diagnosing the disease and determining its complexity or extent in patients with stable coronary artery disease?. Turk. Kardiyol. Dern. Ars..

[40] Turan T., Menteşe Ü., Ağaç M.T., Akyüz A.R., Kul S., Aykan A.Ç., Bektaş H., Korkmaz L., Öztaş Menteşe S., Dursun İ. (2015). The relation between intensity and complexity of coronary artery lesion and oxidative stress in patients with acute coronary syndrome. Anatol. J. Cardiol..

[41] Kharb S. (2003). Low blood glutathione levels in acute myocardial infaction. Ind. J. Med. Sci..

[42] Zuzak E., Horecka A., Kiełczykowska M., Dudek A., Musik I., Kurzepa J., Kurzepa J. (2017). Glutathione level and glutathione reductase activity in serum of coronary heart disease patients. J. Pre-Clin. Clin. Res..

[43] Kaur G., Misra M.K., Sanwal G.G., Shanker K., Chandra M. (1999). Levels of glutathione reductase and glutathione peroxidase of human platelets in unstable angina and myocardial infarction. Boll. Chim. Farm..

[44] Sapira V., Cojocaru I.M., Socoliuc G., Lilios G., Grigorian M., Craiu E., Cojocaru M. (2011). Glutathione reductase levels in patients with unstable angina. Rom. J. Intern. Med..

[45] Lubrano V., Balzan S. (2015). Enzymatic antioxidant system in vascular inflammation and coronary artery disease. World J. Exp. Med..

[46] Gupta S., Sodhi S., Mahajan V. (2009). Correlation of antioxidants with lipid peroxidation and lipid profile in patients suffering from coronary artery disease. Expert Opin. Ther. Targets.

[47] Lubrano V., Di Cecco P., Zucchelli G.C. (2006). Role of superoxide dismutase in vascular inflammation and in coronary artery disease. Clin. Exp. Med..

[48] Kotur-Stevuljevic J., Memon L., Stefanovic A., Spasic S., Spasojevic-Kalimanovska V., Bogavac-Stanojevic N., Kalimanovska-Ostric D., Jelić-Ivanovic Z., Zunic G. (2007). Correlation of oxidative stress parameters and inflammatory markers in coronary artery disease patients. Clin. Biochem..

[49] Violi F., Pignatelli P. (2015). Clinical Application of NOX Activity and Other Oxidative Biomarkers in Cardiovascular Disease: A Critical Review. Antioxid. Redox Signal..

