# Supplementary figures and images for: The Role of Systemic Oxidative Status in Coronary Arterial and Peripheral Venous Blood of Patients with Unstable Angina Pectoris
Source: Life (Basel). 2023 Jul 11;13(7):1537. doi: 10.3390/life13071537 (PMC10381699; doi:10.3390/life13071537)

**Figure S1 CONSORT flow diagram. CONSORT, Consolidated Standards of Reporting Trials.**

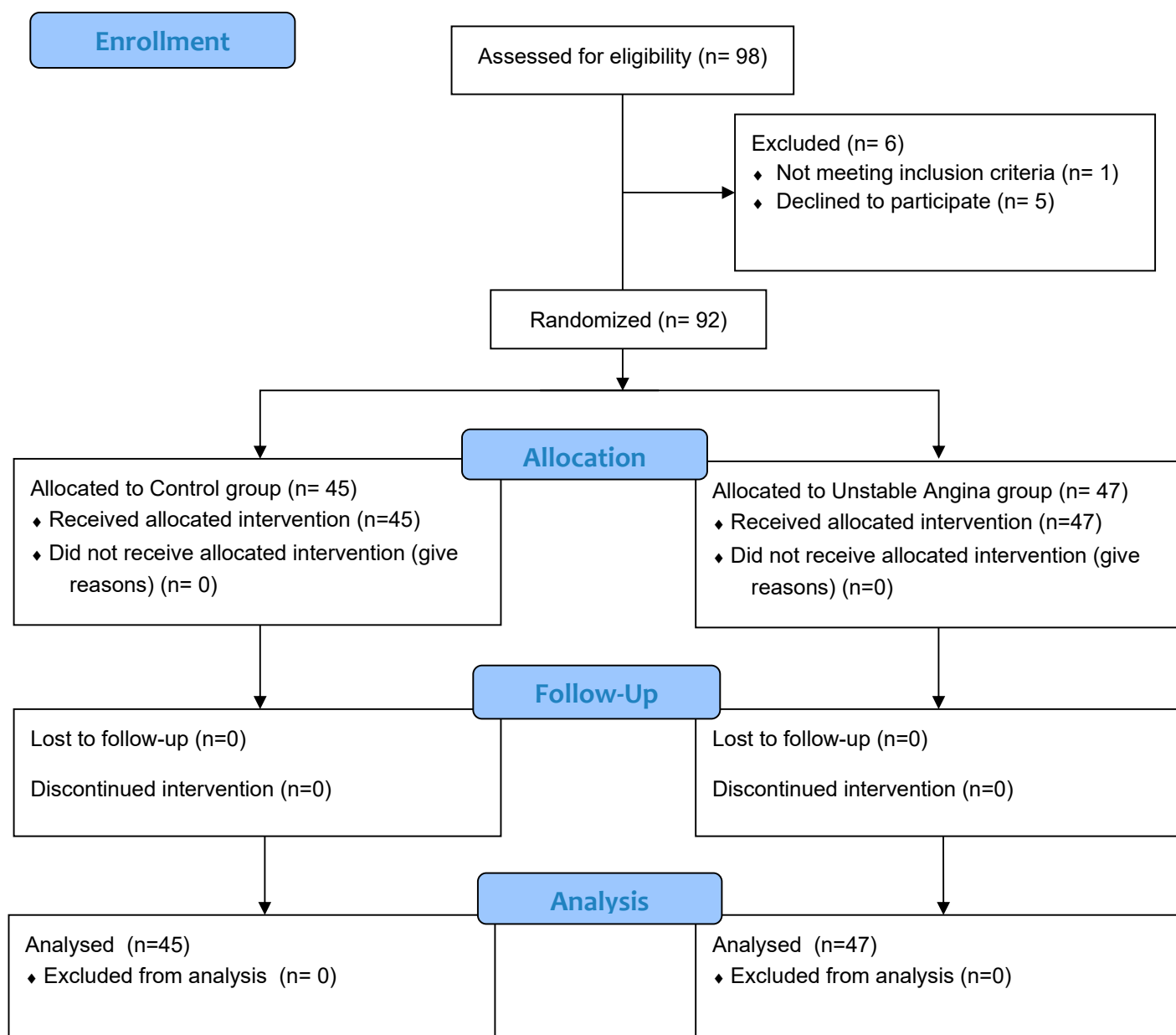

Supplement: Supplementary file 1 [file life-13-01537-s001.zip › life-2467200-supplementary.pdf]
